# LAMP Detection Assays for Boxwood Blight Pathogens: A Comparative Genomics Approach

**DOI:** 10.1038/srep26140

**Published:** 2016-05-20

**Authors:** Martha Malapi-Wight, Jill E. Demers, Daniel Veltri, Robert E. Marra, Jo Anne Crouch

**Affiliations:** 1 United States Department of Agriculture, Agricultural Research Service (ARS), Systematic Mycology and Microbiology Laboratory, Beltsville, MD 20705, USA; 2 Oak Ridge Institute for Science and Education, ARS Research Participation Program, Oak Ridge, TN 37831, USA; 3 Department of Plant Pathology and Ecology, The Connecticut Agricultural Experiment Station, New Haven, CT 06504, USA

## Abstract

Rapid and accurate molecular diagnostic tools are critical to efforts to minimize the impact and spread of emergent pathogens. The identification of diagnostic markers for novel pathogens presents several challenges, especially in the absence of information about population diversity and where genetic resources are limited. The objective of this study was to use comparative genomics datasets to find unique target regions suitable for the diagnosis of two fungal species causing a newly emergent blight disease of boxwood. Candidate marker regions for loop-mediated isothermal amplification (LAMP) assays were identified from draft genomes of *Calonectria henricotiae* and *C. pseudonaviculata*, as well as three related species not associated with this disease. To increase the probability of identifying unique targets, we used three approaches to mine genome datasets, based on (i) unique regions, (ii) polymorphisms, and (iii) presence/absence of regions across datasets. From a pool of candidate markers, we demonstrate LAMP assay specificity by testing related fungal species, common boxwood pathogens, and environmental samples containing 445 diverse fungal taxa. This comparative-genomics-based approach to the development of LAMP diagnostic assays is the first of its kind for fungi and could be easily applied to diagnostic marker development for other newly emergent plant pathogens.

Biological invasions by new and emergent plant pathogens significantly affect crop production and natural environments worldwide (reviewed in^1^). The invasion of boxwood blight disease was first documented during the 1990s in the United Kingdom and New Zealand, and rapidly destroyed wild and commercially grown boxwood (*Buxus* spp.) throughout Europe and western Asia. In 2011, the fungal pathogen responsible for boxwood blight, *Calonectria pseudonaviculata* (=*Cylindrocladium buxicola*) was found in North America, with reports of the disease made almost simultaneously from Connecticut and North Carolina in the eastern U.S.A.^2^. Since then, the disease has been diagnosed across North America in 12 additional states and three Canadian provinces (reviewed in^3^), threatening an industry valued in the U.S.A. alone at $103 million annually. *Calonectria henricotiae*, a second species with increased thermotolerance and reduced sensitivity to fungicides, was recently described from five European countries, increasing the threat to boxwood plants grown in warmer climactic regions^4^. Plants with boxwood blight exhibit necrotic leaf lesions, defoliation, and stem cankers, usually leading to plant death^2^,^5^ (Fig. 1). Infection can be latent, and some evidence suggests that the pathogen may sequester in boxwood cultivars with reduced sensitivity^6^. Because there are no curative treatments—fungicides are at best suppressive of symptoms—infected nursery-grown plants are rendered unfit for sale (*e.g.*^2^,^5^). If plants are not culled from production or destroyed, they provide a long-lived source of inoculum that spreads the pathogen by means of asexual spores or resistant survival structures carried in soil, air, or water^7^. A plant, once infected, can manifest symptoms within several days, and be infective shortly after symptoms appear. Therefore, early diagnosis of infected but asymptomatic plants is crucial to avoidance of disease spread and to implementation of effective disease control strategies.

The development of molecular diagnostic methods has greatly improved the sensitivity, specificity and turnaround time for the identification of plant pathogens, even in the absence of disease symptoms or signs of the causal agent. For newly emergent plant pathogens, the DNA region most commonly used for initial identification purposes is the internal transcribed spacer (ITS) region of the nuclear ribosomal DNA. However, the ITS region may not by itself provide enough resolution to uniquely distinguish among some or all species or sub-specific groups (*e.g.*^8^,^9^,^10^). This can be a particular problem for newly emergent plant pathogens, where information about population and genetic diversity is unavailable or scarce. In the boxwood blight pathosystem, this was the case when the ITS region was used as the basis for a real-time PCR TaqMan tool to quantify *C. pseudonaviculata* from soil, air and water in inoculated trials^7^. Although the ITS-based assay was extremely sensitive (10 fg target gDNA) due to the presence of 76 ITS target copies in the genome, the authors opined that the assay should be used with care, as specificity for the target species was not complete when tested against a sample of four non-target *Calonectria* species^7^. This limitation holds important practical implications, as species in the *Calonectria* genus are ubiquitous, and have the ability to induce disease in nearly 335 plant species from approximately 100 plant families^11^,^12^. *Calonectria* species are also capable of surviving in the soil in a saprophytic or dormant state for many years, even in the absence of a host, and under adverse conditions. Moreover, the *Nectriaceae* family, to which the *Calonectria* genus belongs, comprises many soil-borne saprobes^13^ that have been documented as major constituents of the fungal community in the boxwood rhizosphere^14^. The extent to which such closely related endemic organisms might cross-react with diagnostic tools targeting the boxwood blight pathogens has not yet been determined, but the potential for false positives should be considered in the development of molecular diagnostic tools.

In those instances when the ITS region cannot uniquely identify a pathogen, other DNA targets, often single-copy nuclear housekeeping genes, are investigated for their potential to improve diagnostic specificity^15^,^16^,^17^. However, this approach may result in a trade-off between assay specificity and sensitivity. For example, in the boxwood blight system, Gehesquière and colleagues^7^ reported a four-fold reduction in sensitivity, relative to their ITS assay, using a real-time PCR assay based on beta tubulin 2 (*TUB2*). In addition, although the *TUB2* assay was specific for *C. pseudonaviculata* among a sample of four non-target *Calonectria* species, the possibility for cross-reactivity with non-targets could not be ruled out given the high level of nucleotide similarity in this region of the genome among some *Calonectria* species^7^.

Advances in next-generation DNA sequencing have resulted in the exponential growth of sequenced pathogen genomes^18^, and with it the growth of currently available molecular marker resources, making possible improved surveillance for emerging disease threats. Several viral and bacterial plant pathogens now incorporate next-generation sequencing technology as the basis for diagnostic tool development^19^,^20^,^21^, as the genomes for these organisms are relatively small and tractable. However, the application of genomic-based approaches for the development of fungal plant pathogen diagnostic tools is not yet commonplace. This is likely due to the relatively slower pace of fungal genome sequencing in comparison to that of bacteria and viruses, given the greater complexity and size of fungal genomes, which range from average 19 to 280 Mb 22, and the associated higher costs. But with improved access to fungal genome data through large-scale initiatives such as the 1000 Fungal Genomes Project^18^, the proliferation of sequences generated by individual research groups, and increasing access to biologist-friendly computational tools, fungal pathogen diagnostics will increasingly rely on the power of comparative genomic datasets to improve detection specificity and sensitivity.

In this study, we set out to identify novel, organism-specific marker candidates for the boxwood blight pathogens through comparative genomic analyses of *C. pseudonaviculata, C. henricotiae* and three related species in the *Nectriaceae* at different levels of evolutionary divergence. From a pool of candidate markers, we developed and tested sets of loop-mediated isothermal amplification (LAMP) diagnostic assays, and demonstrated their specificity against a set of fully characterized cultured and environmental samples containing 445 fungal taxa. This comparative genomics approach is the first of its kind applied to fungal pathogens for the development of LAMP diagnostic assays, and the methodology described could be easily applied to other emerging pathogen populations.

## Results

### LAMP primer design and primer validation

To increase the probability of identifying unique targets from the boxwood blight fungi, three different strategies were employed to mine candidate markers from genome datasets. For all three strategies, markers were identified by the alignment of whole draft genomes of *C. henricotiae* CBS 138102, *C. pseudonaviculata* CBS 139707 and three related species from the *Nectriaceae* family that served as outgroup taxa. The outgroup genomes were selected to provide sequence data from different levels of divergence relative to the ingroup genomes, and consisted of *C. naviculata* CBS 101121, *C. leucothoës* CBS 109166 and *Dactylonectria macrodidyma* JAC15-08^23^ (Table 1). Properties of all candidate marker regions and primers tested are summarized in Tables 2 and 3.

For the first marker development strategy (the “unique region” approach), the aim was to identify unique regions present only in the boxwood blight fungi and absent in the outgroup genomes. Twenty sequences that were highly conserved between the *C. pseudonaviculata* and *C. henricotiae* draft genomes and not present in the three outgroup species were randomly identified from the sequence tracks mapped to the genome assembly of *C. pseudonaviculata* CBS 139707. BLASTn and BLASTx searches using 19 of these regions found no significant similarities to any known or characterized sequence in the NCBI database. All 20 candidate marker sequences were identified from intergenic regions and only six of the candidate marker regions were suitable for LAMP primer design. Of the 14 rejected regions, 13 had an A+T ratio >70%, and 57% contained a conserved motif (TTTAT/ATATTATTATTAA/TTTAATA). In contrast, the six candidate marker regions suitable for LAMP primer development had an A+T ratio from 49% to 63%. BLAST analyses against the *C. pseudonaviculata* genome showed that five of the sequences for which primers could be designed were present only once in the genome (Table 2).

The second strategy for identifying candidate markers (the “SNP” approach), focused on regions that were monomorphic within the ingroup but polymorphic between the ingroup and outgroup. Markers were identified from regions with high depth read coverage for *C. pseudonaviculata*, *C. henricotiae*, *C. naviculata*, *C. leucothoës* and *D. macrodidyma* (at minimum, >20–40X coverage), with the additional criterion that they could be aligned between *C. pseudonaviculata* and *C. henricotiae*. Four candidate regions were examined, and four sets of high-scoring LAMP primers were designed from three of the regions meeting these criteria. The fourth region was rejected because of ingroup/outgroup polymorphisms (data not shown). BLASTx searches of the NCBI database showed that, of the three selected target regions, one region likely encodes a protein in the patatin-like phospholipase superfamily, while the other two regions encode proteins whose functions could not be predicted. All of the targeted regions were single copy within the *C. pseudonaviculata* and *C. henricotiae* genomes (Table 2).

The third marker development strategy (the “bioinformatics” approach) employed a computational workflow to identify candidate marker sequences. A series of sequence alignments were used to identify regions sharing high sequence identity between *C. pseudonaviculata* and *C. henricotiae* but low sequence identity to the non-target outgroup genomes. Of the 24 candidate sequences selected by the pipeline, two were rejected as poor primer targets. Running BLASTx using the non-redundant database returned seven targets with no hits, and a wide-range of results for the other sequences (Table 2).

Altogether, 32 candidate markers were identified and examined for specificity using the three different marker development strategies (Supplementary Table S1). First, primers were evaluated by conventional PCR reactions using the outer primers F3 and B3 to assess amplicon production and by using gDNA from three non-target organisms—*C. chinensis*, *D. macrodidyma*, and *Trichoderma harzianum*—to broadly assess specificity. Second, each of the 32 candidate markers was evaluated in conventional LAMP reactions using the full set of six primers per set. *Calonectria pseudonaviculata* CBS 139707 and water were used as positive and negative controls, respectively, for both evaluations (Supplementary Table S1). Primers used in these tests are listed in Tables 3 and S2. These preliminary analyses resulted in four primers sets—P.17, P.38, P.25.4 and P.24—with desirable characteristics for diagnostics, in that they: (i) did not show false positive amplifications with the outgroups tested; (ii) consistently resulted in positive/bright bands amplified from the positive control; and (iii) produced neither background nor amplification, caused by primer dimerization, from the negative control (summarized in Supplementary Table S1). To further assess the robustness of P.17, P.38, P.25.4 and P.241 primers, gDNA extracted from a defined, heterogeneous mixture of fungi present in boxwood environments was evaluated; no false positives were observed. *Volutella pachysandra* and *V. buxi* were also tested since they are economically important and widespread pathogens and endophytes of the *Buxaceae* family^24^,^25^,^26^; no false positive amplifications were observed when gDNA of these species was screened using the four LAMP primer sets (Table 1, Supplementary Table S1). Finally, following sensitivity evaluation using *C. pseudonaviculata* gDNA as the control, primer sets P.38, P.25.4 and P.241 were chosen for full testing for identification of the boxwood blight fungi.

### LAMP assay specificity and sensitivity

Primer set P.38 targets a unique 539 bp non-coding sequence that showed 100% identity between *C. pseudonaviculata* and *C. henricotiae* and was not present in the outgroup genomes. Using the Conserved Domain Database (CDD) search^27^,^28^, the putative genes located at the 5′ and 3′ ends of the marker were predicted to possess epoxide hydrolase and glycosil transferase domains, respectively. Analysis of the primer set P.25.4, which targets a patatin-like phospholipase, showed that the binding sites were highly conserved between *C. pseudonaviculata* and *C. henricotiae*, with only two single-nucleotide polymorphisms (SNPs) among them. The primer binding sites were polymorphic compared to the outgroup species: 44 SNPs were observed between *C. pseudonaviculata* and *C. naviculata*, and 44 SNPs were observed between *C. pseudonaviculata* and *C. leucothoës*. This sequence was not found in the *D. macrodidyma* genome. Alignment of the corresponding region of P.241 revealed 99.3% shared sequence identity between *C. pseudonaviculata* and *C. henricotiae*. A BLASTx analysis identified the region as part of a predicted protein containing a conserved RNA recognition motif.

**Figure 2:**
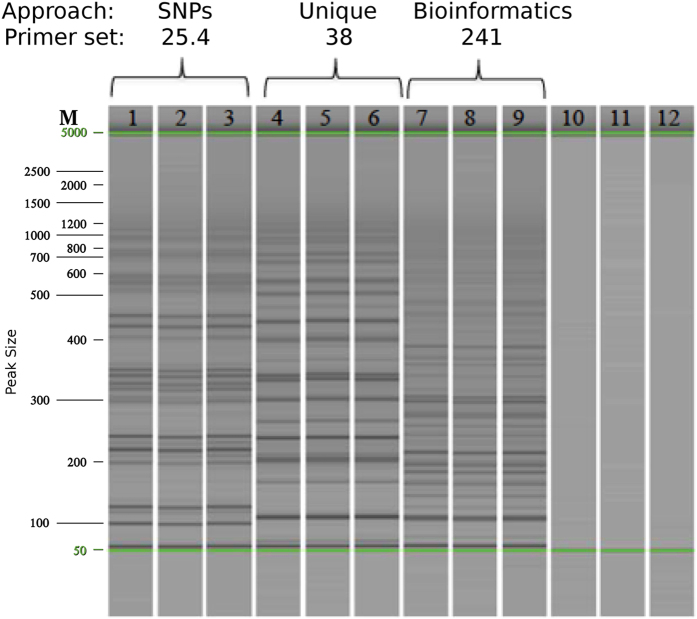
LAMP detection of *Calonectria henricotiae* and *C. pseudonaviculata* isolates using three different primers sets. Note the different patterns of ladder-like fragments from each set. Lanes: M: DNA marker; 1–9: *C. pseudonaviculata* and *C. henricotiae* isolates; 10–12: negative controls.

To assess the robustness of the three LAMP primer sets, 20 isolates of *C. pseudonaviculata* and *C. henricotiae* belonging to the most common simple sequence repeat genotypes (G1MG1 – G1MG4 and G2)^29^ and from a wide range of geographic regions were tested (Table 1). LAMP assays of these 20 isolates using the three primer sets showed clear and consistent positive results for all samples evaluated, and distinctive patterns of ladder-like fragments were observed from each primer set (Fig. 2). No amplifications were observed when DNA was not added to the assays. Next, 11 additional *Calonectria* spp. isolated from soil and different host plants were examined using the assays (Table 1). No cross-reactivity was observed in reactions with primer sets P.38 and P.25.4. However, primer set P.241 amplified DNA from *C. densa* and *C. pini*, but not from the other nine *Calonectria* spp. assessed in this study. To further test the specificity of the LAMP assays, 18 boxwood rhizosphere samples from the U.S. National Arboretum (USNA) were evaluated. These rhizosphere samples contained a diverse collection of 445 fungal taxa, including species of *Bionectria*, *Cyanonectria*, *Cylindrocarpon, Chaetomium, Dactylonectria*, *Fusarium*, *Nectria* and *Trichoderma*^14^. No false positive results were obtained from the three LAMP sets (Table 4) when used to screen these samples, confirming the robustness of these assays.

To determine the sensitivity of the LAMP assays, primer sets were tested against serial dilutions of *C. pseudonaviculata* CBS 139707 gDNA that were prepared from concentrations determined using a Qubit 2.0 fluorometer (Life Technologies). The detection limit was 100 pg per reaction for all three assays.

## Discussion

This study represents the first use of a comparative genomics approach to design LAMP diagnostic assays for fungi^30^. We used three different comparative-genomics-based approaches to locate suitable target regions for LAMP-based detection assays specific to the two *Calonectria* fungal species that cause boxwood blight disease. From a practical standpoint, our goal was the development of LAMP assays capable of rapid identification of boxwood blight fungi by diagnostic clinics and labs without ready access to thermal cyclers or other specialized equipment^31^,^32^,^33^. Although we chose to design LAMP-based detection assays, the candidate markers identified in this study could also be used as the basis of real-time PCR or other wet-lab detection technology, as preferred by the end user. The greater ease of use and fewer equipment requirements of LAMP compared to real-time PCR make these assays more amenable to use in diagnostic laboratories or for further development into a commercially available diagnostic kit. In the present study, we made use of a capillary gel electrophoresis unit and other sophisticated tools to benchmark the assays; however, a minimal set of tools—a water bath and standard gel electrophoresis equipment—could be used with equally effective results in a diagnostic laboratory setting. Implementation of the assays might be further simplified by using a visual indicator for closed tube end-point detection rather than electrophoresis (*e.g.* the addition of hydroxynaphthol blue, calcein, berberine, gold nanoparticles, SYBR green overlaid onto agar), minimizing the chance of contamination by eliminating the need to open the assay tubes. At present, many colorimetric-based visualization methods yield unreliable data, or are subject to visual misinterpretation, but active research in the area indicates several promising advances for the visualization of LAMP products (*e.g.*^34^,^35^).

The three LAMP assays developed in this study were all able to detect a minimum of 100 pg of DNA, which is similar to the detection limits reported for several other LAMP assays for fungi^30^. The targeted gene regions are all single copy within the *Calonectria* genomes, which decreases the sensitivity of the assays compared to multi-copy regions, *e.g.* ITS, which is estimated to have 76 copies in *C. pseudonaviculata*^7^. Targeting single-copy regions, however, improved the specificity of the assay compared to a real-time assay based on the ITS region^7^, enabling specific detection of the two *Calonectria* species responsible for boxwood blight disease.

In the process of ensuring the specificity of new diagnostic tools, it is critical that inadvertent cross-reactivity with non-target microorganisms inhabiting the host environment be taken into account and avoided. Community assessments of the boxwood rhizosphere using pyrosequencing have identified 445 fungal taxa^14^, which include a large and abundant cohort of closely related species that could potentially react with assays, thereby producing false positives. We observed no false positives when the three LAMP assays were used to test the environmental DNA extracted from those 445 fungal taxa, nor did we observe false positives from closely related congeneric species. Importantly, the three LAMP assays identified all known *C. pseudonaviculata* and *C. henricotiae* simple sequence repeat genotypes^36^ originating from across the United Kingdom, Europe, western Asia and North America.

Each of the three comparative-genomics-based approaches employed in the current study presented advantages and disadvantages for the identification of specific candidate marker regions. The “bioinformatics approach,” the only one to involve a high-throughput strategy, relied solely on a computational pipeline. For downstream applications where more than a single marker is needed, or where genome-wide datasets are required, this approach is advantageous in that it is much more likely to provide a larger cohort of candidate markers in a more reasonable time frame, relative to the “unique regions” and “SNP-based” approaches, which relied on initial manual assessments of candidate sequences *in silico*. The bioinformatics approach also had the advantage of eliminating potential sources of ascertainment bias, by treating all sequence data as equal based on *ab initio* parameters, without regard to whether or not the candidate region was situated in a gene, intron, or intergenic region. However, none of the 23 tested candidate markers derived from the computational pipeline yielded 100% target-specific markers when they were screened against biological samples in the wet lab. Performance of this method might be improved through optimization of parameters or increased evaluation of genome space. The candidate marker regions obtained from the computational pipeline were identified from only a single contig in *C. pseudonaviculata* CBS 139707, and all returned at least one significant alignment hit with a non-target *Calonectria* species. However, if all of the contigs from the organismal genomes were evaluated and exhaustively searched, it is possible that superior candidates could be found whose sequences have low or no similarity to those from non-target taxa, when performing alignments with FASTA or a similar program like BLAST. Performance of the method could also be improved by including additional genomes into the group of non-target taxa, use of a more stringent E-value, or different identity cutoff settings.

The “SNP-based” approach for candidate marker identification, where highly variable regions of the genome were targeted through a combination of variant calls and visual examinations, is an extension of a well-established practice used by researchers for the design of diagnostic assays based on housekeeping gene sequences^37^. The use of SNP-based markers is increasingly important across many fields of biological study^38^, with SNPs forming the basis of many LAMP diagnostic assays for fungal pathogens, especially within the rDNA ITS region and the *TUB2* gene^30^. This strategy has the advantage that the differences between the target organism and the outgroup taxa are known with certainty. However, because the targeted region is found in all species, the assay is more likely to yield false positives from closely related taxa. In the present study, amplification of the SNP-based targets was highly specific for three of the four tested LAMP primer sets, although only one primer set produced consistently clear products when visualized using capillary electrophoresis.

The “unique regions” approach was used to mine candidate markers from regions of the genome that were unique to *C. pseudonaviculata* and *C. henricotiae*, relative to the three outgroup species. In contrast with the “SNP-based” approach, we predicted that focusing on lineage-specific sequences would produce highly specific LAMP markers that would not cross-react with any non-target species. Identification of candidate marker regions absent in the non-target organisms required a sufficiently high depth of read coverage to ensure that the sequence was truly absent, rather than simply missing from the genome assembly due to low coverage. Although we did not specifically target intergenic regions, 19 of the randomly selected, lineage-specific candidate markers identified using this approach mapped within intergenic regions. Seventy percent of the original 20 candidates were excluded from consideration because high A+T content (>70%) and low complexity precluded efficient LAMP-specific primer design. However, even with the need to exclude most of the original candidate regions, the “unique regions” approach yielded the best success rate, with three of the resulting LAMP assays providing highly specific, reproducible detection of the targeted boxwood blight fungi.

Although the application of comparative genomics for diagnostic tool development is not yet commonplace for fungi, this approach is increasingly prevalent for pathogenic organisms with smaller and therefore more tractable genomes. Virologists and bacteriologists are using several novel genome-based strategies, including comparative-genomics pipelines for the diagnosis of pathogens (reviewed in^39^,^40^) using publicly available genome sequences^20^,^41^,^42^,^43^, to develop conventional PCR and LAMP assays of superior specificity^20^,^41^. To our knowledge, this is the first study using a comparative-genomics-based approach to develop a LAMP diagnostic assay for the identification of fungal plant pathogens. The data presented here demonstrate the specificity of LAMP assays designed to exclusively detect the two species of *Calonectria* pathogens associated with boxwood. The assays developed in this study should provide the foundation for monitoring and development of mitigation strategies aimed at controlling the two fungal pathogens that induce boxwood blight.

## Methods

### Biological samples and DNA extraction

Fungal cultures used in this study (summarized in Table 1) included a total of 13 cultures of *C. pseudonaviculata* and seven cultures of *C. henricotiae* originally isolated from blighted boxwood plants. To test the specificity of LAMP assays against target and also non-target species, 11 representative isolates of different *Calonectria* species were evaluated. Cultures of additional fungi known as ubiquitous colonizers of boxwood plants were also included (Table 4). Fungi were grown on V8 agar (200 ml V8 juice l^−1^, 3 g CaCo3 l^−1^, and 20 g agar l^−1^) or potato dextrose agar plates (PDA; Fisher Scientific, Pittsburg, PA).

To test whether members of the endemic fungal community associated with boxwood would cross-react with the diagnostic assays, 19 sets of biological samples were evaluated. First, we extracted total genomic DNA from fresh boxwood leaves collected from the United States National Arboretum (USNA; Washington, D.C.)^44^, which included both the leaf and associated endophytic fungal symbionts. Leaves were stored at −20 °C until use. The second set of samples comprised total genomic DNA extracted from the rhizosphere of 18 boxwood plants at the USNA. These rhizosphere samples contained a diverse collection of 445 fungal genera, as characterized using pyrosequencing of the ITS region on the Roche 454 platform (Roche Lifescience, Nutley NJ)^14^.

Fungal gDNA was extracted as previously reported^23^. Nucleic acids were quantified using a Qubit 2.0 fluorometer (Life Technologies, Grand Island, NY), and subsequently normalized for use in LAMP reactions. *Calonectria henricotiae* and *C. pseudonaviculata* isolates used in this study were identified to genus level by Sanger sequencing of the *TUB2* region generated by PCR using the primer pair Bt2a/Bt2b^45^ and to the species level through multi-locus sequence analysis as described^7^.

### Identification of candidate marker regions and LAMP primer design

Candidate marker regions for LAMP assay design were identified based on the alignment of whole draft genomes of *C. pseudonaviculata* CBS 139707 and *C. henricotiae* CBS 138102 (data not shown). Genome sequence assemblies of three related species at different levels of divergence from the boxwood blight pathogens were used for comparative analyses: *C. naviculata* CBS 101121 isolated from leaf litter; *C. leucothoës* CBS 109166 isolated from *Theobroma cacao*; and the plant pathogen *D. macrodidyma*^23^. In order to increase the chance of finding species-specific diagnostic targets, three different approaches were used to mine the genome datasets. For the first two approaches, CLC Genomics Workbench software v.7.0 (CLC bio, Boston, MA) was used to identify candidate marker sequences that differed between target organisms (*C. pseudonaviculata* and *C. henricotiae*) and non-target organisms (*C. naviculata*, *C. leucothoës* and *D. macrodidyma*). A consensus sequence was generated from *C. naviculata, C. leucothoës* and *D. macrodidyma* using a threshold value of 0 and by inserting an N ambiguity symbol in low coverage regions. The consensus sequence was mapped back against the reference *C. pseudonaviculata* CBS 139707 draft genome assembly. Trimmed Illumina sequence reads for all five species were mapped to the *C. pseudonaviculata* CBS 139707 assembly using a mismatch cost of 1 bp, a deletion and insertion cost of 2 bp, and a length and similarity fraction of 0.5. Mapped data were visualized as tracks in CLC Genomics. For the identification of candidate markers, our first approach (the “unique region” approach) used these mapping files to visually identify regions that were uniquely present in *C*. *pseudonaviculata* and *C. henricotiae*, but not in the other three genome assemblies, using CLC Genomics track tools. Our second approach (the “SNP” approach) used the CLC Genomics Workbench to identify regions that were present and monomorphic within the boxwood blight fungi, but contained numerous SNPs in comparison with the outgroups. Nucleotide polymorphisms between the reference genome and mapped species were identified using the CLC Genomics Workbench Probabilistic Variant tool, using a cut-off of 95% probability, and ignoring non-specific regions. Variant tracks were added to the reference genome assembly tracks and each candidate marker visually inspected. Targeted regions were selected that were not polymorphic between *C. pseudonaviculata* and *C. henricotiae*, but were polymorphic between *C. pseudonaviculata* and non-target *Calonectria* species, particularly at the 3′ end of the F3 and B3 primers and at the 5′ end of the FIP and BIP primers. To find candidate markers based on the unique region and SNP approaches, selected targets were >400 bp, did not fall on the end of contigs or areas with read errors, and had sufficient coverage (at minimum, >20–40X coverage).

The third approach (the “bioinformatics” approach) employed a computational workflow to identify candidate marker sequences. A series of sequence alignments was used to find sequence fragments sharing high sequence identity between *C. pseudonaviculata* and *C. henricotiae* but low sequence identity to the non-target genomes *C. naviculata, C. leucothoës* and *D. macrodidyma*. The first step aligned contig 149 in *C. pseudonaviculata* to contig 1 in *C. henricotiae* using MUMmer v.3.23^46^ under default settings. Matching alignments were then split into fragments 539 bp long, and those fragments sharing ≥98% sequence identity were saved into a collection of candidate marker regions. Pairwise sequence alignment between candidate markers and all contigs of non-target taxa was then performed using FASTA v. 36^28^ under default settings. The top bit score among hits for each candidate marker was saved, and candidates were then sorted from low to high score (ordered by least to most similarity with the non-target species). A total of 24 candidate regions with bit scores ranging from 57.6 to 864.2 were chosen for further evaluation with LAMP primer design software.

LAMP primers were designed using the LAMP Designer software v.1.10 (Premier Biosoft, CA, USA). Primer sets were selected based on the criteria described by Notomi and colleagues^31^ and consisted of six oligonucleotide primers, including two outer primers (F3 and B3), two inner primers (FIP and BIP) and two loop primers (LF and LB). Primers were synthesized by Integrated DNA Technologies (IDT, Coralville, IA). Gene models were identified from target contigs using the AUGUSTUS web server, with *Fusarium graminearum* as the model for predictions^47^. Putative gene functions were identified where possible using BLAST searches of the NCBI GenBank databases (http://www.ncbi.nlm.nih.gov/genbank/).

### LAMP assay

LAMP reactions were performed using the OmniAmp^TM^ RNA & DNA LAMP kit (Lucigen Corporation, Middleton, WI) following the manufacturer’s protocol. Briefly, each 12.5 μl reaction consisted of 1 μl of gDNA, 1.25 μl of 10× DNA Polymerase Buffer C, 0.4 μl of 25 mM dNTPs, 1 μl of 100 mM MgSO_4_, 0.4 μl of 5M betaine, 0.25 μl of OmniAmp DNA Polymerase and 1.25 μl of 100 μM primer mix. The LAMP primer mix consisted of 2 μM each of the F3 and B3 primers, 8 μM each of the LF and LB primers, and 16 μM each of the FIP and BIP primers. Reactions were incubated as recommended by the manufacturer at 70 °C for 30 min in a C1000 Thermal Cycler (Bio-Rad Laboratories Inc., Hercules, CA). LAMP reactions were performed with templates of 1 ng of fungal gDNA and 10 ng of total DNA isolated from boxwood environmental samples. DNA from *C. pseudonaviculata* CBS 139707, which was used as a genome reference for the design of primers, served as a positive control. Amplifications were assessed on a QIAxcel capillary gel electrophoresis instrument system (QIAGEN, Germantown, MD) using the QIAxcel DNA screening cartridge (QIAGEN). Reactions were performed a minimum of two times for all samples tested.

## Additional Information

**Accession codes:** GenBank accession numbers KT885902–KT885932.

**How to cite this article**: Malapi-Wight, M. *et al*. LAMP Detection Assays for Boxwood Blight Pathogens: A Comparative Genomics Approach. *Sci. Rep.* **6**, 26140; doi: 10.1038/srep26140 (2016).

## Supplementary Material

Supplementary Information

## Figures and Tables

**Figure 1:**
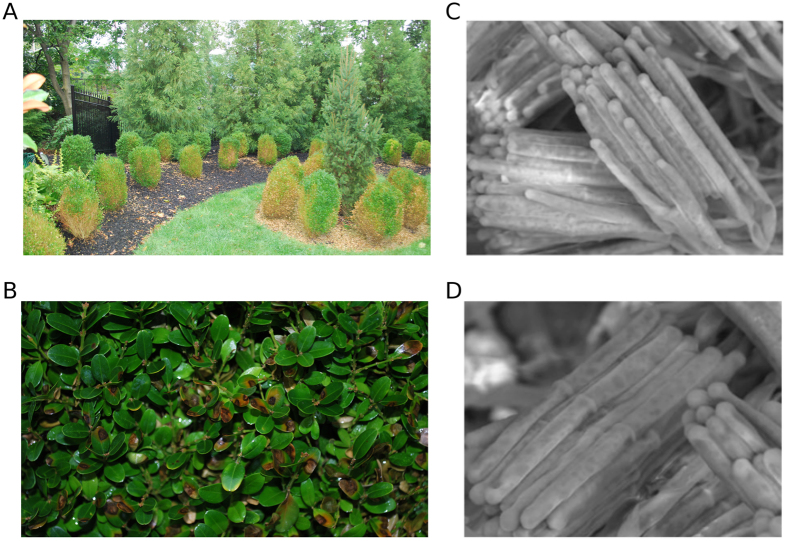
Images of *Calonectria pseudonaviculata* and *C. henricotiae*. **(A,B)** Infection of *C. pseudonaviculata* on a boxwood landscape in New Jersey (images courtesy of Richard Buckley). **(C,D)** Scanning electronic images of *C. henricotiae* conidiophores and cylindrical conidia.

**Table 1:** Isolates of *Calonectria* and other fungal species used to test the LAMP primer sets for specificity to the target species, *C. henricotiae* and *C. pseudonaviculata*.

Species	Isolate name	Host origin	Geographic origin	Detection usingLAMP primer setsP. 38 P.25.4 P. 241		
*C. pseudonaviculata*						
	Bdv B/52-3905	*Buxus* sp.	Switzerland	+	+	+
	CBS 139707 (aka cpsCT1)	*Buxus* sp.	USA	+	+	+
	CpsCT13	*Buxus* sp.	USA	+	+	+
	MM2013calTA8	*B. colchica*	Iran	+	+	+
	MM2013 calTA12	*B. colchica*	Iran	+	+	+
	PD011/05318376	*B. sempervirens* ‘Suffruticosa’	The Netherlands	+	+	+
	PD011/05461949	*Buxus* sp.	The Netherlands	+	+	+
	RHS10868	*B. sempervirens* ‘Suffruticosa’	United Kingdom	+	+	+
	RHS 190943	*Buxus* sp.	United Kingdom	+	+	+
	TU005	*Buxus* sp.	Turkey	+	+	+
	30030	*Buxus* sp.	United Kingdom	+	+	+
	11-416-11	*B. sempervirens*	Slovenia	+	+	+
	91.9.6B	*B. colchica*	Iran	+	+	+
*C. henricotiae*						
	CB098	*B. sempervirens*	Belgium	+	+	+
	CBS 138102	*B. sempervirens*	Belgium	+	+	+
	NL009	*B. sempervirens*	The Netherlands	+	+	+
	NL016	*B. sempervirens*	The Netherlands	+	+	+
	NL017	*B. sempervirens*	The Netherlands	+	+	+
	P-10-5782	*Buxus* sp.	Germany	+	+	+
	08-442	*B. sempervirens*	Slovenia	+	+	+
*Calonectria* spp.						
* C. angustata*	CBS 109065	*Tillandsia capitata*	FL, USA	−	−	−
* C. chinensis*	CBS 114827	Soil	Hong Kong	−	−	−
* C. colombiana*	CBS 115127	Soil	Colombia	−	−	−
* C. densa*	CBS 125250	Soil	Ecuador	−	−	+
* C. gracilipes*	CBS 111141	*Eucalyptus globulus*	Colombia	−	−	−
* C. ilicicola*	CBS 115897	*Anacardium* sp.	Brazil	−	−	−
* C. leucothoës*	CBS 109166	*Leucothoe axillaris*	FL, USA	−	−	−
* C. madagascariensis*	CBS 114571	Soil	Madagascar	−	−	−
* C. multiphialidica*	CBS 112678	Soil	Cameroon	−	−	−
* C. naviculata*	CBS 116080	Soil	Brazil	−	−	−
* C. pini*	CBS 125523	*Pinus patula*	Colombia	−	−	+
Other fungal species						
* Dactylonectria macrodidyma*	JAC15-08	−	−	−	−	−
* Trichoderma harzianum*	GJS09-1536	*Theobroma cacao*	Peru	−	−	−
* Volutella pachysandra*	AR2822	*Buxus* sp.	Maryland, USA	−	−	−
* Volutella buxi*	AR2711	*Buxus* sp.	Maryland, USA	−	−	−

Results of three LAMP assays are indicated as giving a positive (+) or negative (−) reaction, based on a minimum of two replicates.

**Table 2:** Summary of the characteristics of the *Calonectria henricotiae* and *C. pseudonaviculata* genome regions used to design LAMP markers in this study.

No.	Primer set/Approach	Marker	Length (bp)	No. copies/genome	BLASTX prediction	GC%	GenBank Accession No.
	Unique						
1	P.1	Intergenic	2313	Multiple	DNA transposase	37	KT885902
2	P.12	Intergenic	507	Single	N/A	43	KT885903
3	P.17	Intergenic	554	Single	N/A	40	KT885904
4	P.38	Intergenic	540	Single	N/A	48	KT885905
5	P.44	Intergenic	501	Single	N/A	51	KT885906
6	P.44B	Intergenic	964	Single	N/A	46	KT885907
	SNPs						
7	P.C1	Coding region	532	Single	Hypothetical protein	51	KT885908
8	P.20	Coding region	1604	Single	Hypothetical protein	53	KT885909
9	P.25.1	Coding region	1173	Single	Patatin and cPLA2 superfamily	50	KT885910
10	P.25.4	Coding region	1173	Single	Patatin and cPLA2 superfamily	50	KT885910
	Bioinformatics						
11	P.119	Intergenic	539	Single	N/A	50	KT885911
12	P.152	Intergenic	539	Single	4f5 domain	57	KT885912
13	P.241	Coding region	539	Single	RNA recognition motif	58	KT885913
14	P.267	Coding region	539	Single	Hypothetical protein	69	KT885914
15	P.267-S13	Intergenic	539	Single	Hypothetical protein	52	KT885915
16	P.269	Intergenic	539	Single	N/A	49	KT885916
17	P.304	Coding region	539	Single	N/A	57	KT885917
18	P.329	Intergenic	539	Single	Hypothetical protein	45	KT885918
19	P.344	Intergenic	539	Single	N/A	54	KT885919
20	P.379	Intergenic	539	Single	Golgi matrix protein	53	KT885920
21	P.381	Intergenic	539	Single	N/A	58	KT885921
22	P.393	Coding region	539	Single	Serine threonine rich protein	59	KT885922
23	P.402	Coding region	539	Single	Hypothetical protein	59	KT885923
24	P.420	Coding region	539	Single	N/A	56	KT885924
25	P.433	Coding region	539	Single	Golgi matrix protein	50	KT885925
26	P.454	Coding region	539	Single	Protein penyltransferase	57	KT885926
27	P.465	Coding region	539	Single	Golgi matrix protein	58	KT885927
28	P.531	Intergenic	539	Single	*CHD5* domain	59	KT885928
29	P.662	Intergenic	539	Single	Hypothetical protein	49	KT885929
30	P.691	Intergenic	539	Single	N/A	53	KT885930
31	P.747	Intergenic	539	Single	Hypothetical protein	55	KT885931
32	P.864	Intergenic	539	Single	Hypothetical protein	51	KT885932

N/A = not available.

**Table 3:** List of LAMP primers used in this study.

Primer sets	Primer^1^	Primer name	Sequence (5′→ 3′)	Tm	GC%
P.38	F3	C.38-F3	CAGTATGACGAGCGGATC	59.4	55.6
	B3	C.38-B3	AAATTTGGCGGTTGTGATTG	60.2	40.0
	FIP	C.38-FIP	CGGTTGATCGCCATTGCATTA GAACGGCATACGAGACAATC	66.5	48.8
	BIP	C.38-BIP	AACAAGGGTCCGTCTGCGAA CTCAGACTGTTGGAATGG	67.8	52.6
	loopF	C.38-LF	CCATATGATTCGGTACGTCCTT	62.0	45.5
	loopR	C.38-LB	GATCCGCAGTCCTCATTCC	62.3	57.9
P.25.4	F3	25.4-F3	GTCAGGCTTCTTCAGATAATCT	59.7	40.9
	B3	25.4-B3	CCTCAGACTCCTGGCTAA	59.7	55.6
	FIP	25.4-FIP	GACAGCGAGACGAGCATAAGA ATCTTCCAATCCGATAGGCT	66.3	48.8
	BIP	25.4-BIP	GACCCAACATGTGTCGGGAAG CATCGTCTGTAATGGACTT	70.0	49.2
	loopF	25.4-LF	TTCAACCCTCCTCTTGGAAAG	60.2	47.6
	loopR	25.4-LB	TTGCGTTGGTGTTGGTTG	61.5	50.0
P.241	F3	241-F3	CGGCTTGATGTCAGGAATT	60.2	47.4
	B3	241-B3	GACACATCCTCAAGTGCC	60.1	55.6
	FIP	241-FIP	CCAGCGACTTGACAAGCCTGCT TGTCCTGTTCCTTGG	68.8	56.8
	BIP	241-BIP	CATCTTGTTCCAGGCGACCTCA CGACGACCTATTCAAGG	67.3	53.8
	loopF	241-LF	CCTGGGCTACCAAGGTCT	62.8	61.1
	loopR	241-LB	TCTTGAATCGTCGGTTGGC	62.8	52.6

^1^F3: Forward outer; B3: reverse outer; FIP and BIP: inner LAMP primers; loopF and loopR: forward and reverse loop primers.

**Table 4:** Environmental samples from boxwood rhizosphere tested using LAMP diagnostic assays for *Calonectria henricotiae* and *C. pseudonaviculata*. Results of the three LAMP assays are indicated as giving a positive (+) or negative (−) reaction, based on a minimum of two replicates.

Samplename	Host rhizosphere	No. fungal taxa in sample	Geographic origin	LAMP primer setsP. 38 P.25.4 P. 241		
VA7040H	*Buxus sinica* var. *insularis*	101	Virginia, USA	−	−	−
VA33904H	*B. sinica* var. *insularis* ‘Justin Brouwers’	80	Virginia, USA	−	−	−
VA 26389H	*B. microphylla* ‘Curly Locks’	92	Virginia, USA	−	−	−
330904H	*B. sinica* var. *insularis* ‘Justin Brouwers’	88	Washington, D.C., USA	−	−	−
51906H	*Buxus* ‘Green Mound’	79	Washington, D.C., USA	−	−	−
29703H	*B. sempervirens* ‘Suffruticosa’	99	Washington, D.C., USA	−	−	−
18834H	*B. harlandii*	63	Washington, D.C., USA	−	−	−
51896H	*B. wallichiana*	56	Washington, D.C., USA	−	−	−
4227R	*B. microphylla* var. *japonica*	66	Washington, D.C., USA	−	−	−
4210H	*B. sempervirens* ‘Joe Gable’	85	Washington, D.C., USA	−	−	−
6395	*B. sempervirens* ‘Vardar Valley’	84	Washington, D.C., USA	−	−	−
4899CH	*B. microphylla* ‘Compacta’	64	Washington, D.C., USA	−	−	−
4220J	*B. sempervirens* ‘Elegantissima’	92	Washington, D.C., USA	−	−	−
51905J	*Buxus ‘*Green Mountain’	80	Washington, D.C., USA	−	−	−
51910K	*B. sempervirens* ‘Northland’	60	Washington, D.C., USA	−	−	−
51904K	*Buxus* ‘Green Gem’	80	Washington, D.C., USA	−	−	−
33789L	*B. sempervirens* ‘Graham Blandy’	80	Washington, D.C., USA	−	−	−
36365K	*Buxus colchica*	80	Washington, D.C., USA	−	−	−

Results of the three LAMP assays are indicated as giving a positive (+) or negative (−) reaction, based on a minimum of two replicates.
