# Supplementary material for: LAMP Detection Assays for Boxwood Blight Pathogens: A Comparative Genomics Approach
Source: Sci Rep. 2016 May 20;6:26140. doi: 10.1038/srep26140 (PMC4873745; doi:10.1038/srep26140)
Supplement: Supplementary Information [file srep26140-s1.doc]

**LAMP Detection Assays for Boxwood Blight Pathogens: A Comparative Genomics Approach**

Martha Malapi-Wight, Jill E. Demers, Daniel Veltri, Robert E. Marra, and Jo Anne Crouch

**Supplementary Table S1.** Fungal samples used for the first round of specificity testing using conventional PCR with outer primers F3 and B3 and LAMP reactions. Results of the PCR assays are indicated as giving a positive (+) or negative (−) reaction, based on a minimum of two replicates.

| **Primer set/**  **Approach** | ***Calonectria pseudonaviculata* CBS 139707^1,2^** | **H_2_0^1,2^** | ***Calonectria chinensis***  **CBS 114827^1^** | ***Dactylonectria macrodidyma*JAC15-08^1^** | ***Trichoderma harzianum***  **GJS09-1536^2^** | ***Colletotrichum fioriniae* ^2,3^**  **MA8-1A2** | ***Volutella pachysandra* AR2822^2^** | ***Volutella buxi***  **AR2711^2^** |
| --- | --- | --- | --- | --- | --- | --- | --- | --- |
| **Unique** |  |  |  |  |  |  |  |  |
| P.1 | + ^NC^ | − | − | − | − | N/A | N/A | N/A |
| P.12 | + ^NC^ | − | − | − | − | N/A | N/A | N/A |
| P.17 | + | − | − | − | − | − | − | − |
| **P.38** | **+** | − | − | − | − | − | − | − |
| P.44 | + ^W^ | − | − | − | − | N/A | N/A | N/A |
| P.44B | + | + | − | − | − | N/A | N/A | N/A |
| **SNPs** |  |  |  |  |  |  |  |  |
| P.C1 | + ^NC^ | − | − | − | − | N/A | N/A | N/A |
| P.20 | + ^W^ | − | − | + | − | N/A | N/A | N/A |
| P.25.1 | + ^W^ | − | − | − | − | N/A | N/A | N/A |
| **P.25.4** | **+** | − | − | − | − | − | − | − |
| **Bio-informatics** |  |  |  |  |  |  |  |  |
| P.119 | + | − | − | − | + | N/A | N/A | N/A |
| P.152 | + ^NC^ | − | − | − | − | N/A | N/A | N/A |
| **P.241** | **+** | − | − | − | − | − | − | − |
| P.267 | + | − | − | + | + | N/A | N/A | N/A |
| P.267-S13 | + | + | − | − | − | N/A | N/A | N/A |
| P.269 | + ^NC^ | − | − | − | − | N/A | N/A | N/A |
| P.304 | + | − | − | + | + | N/A | N/A | N/A |
| P.329 | + ^NC^ | − | − | − | − | N/A | N/A | N/A |
| P.344 | + ^NC^ | − | − | − | − | N/A | N/A | N/A |
| P.379 | + | − | − | − | + | N/A | N/A | N/A |
| P.381 | + | + | − | − | + | N/A | N/A | N/A |
| P.393 | + | − | + | + | + | N/A | N/A | N/A |
| P.402 | + | − | − | − | + | N/A | N/A | N/A |
| P.420 | + | − | + | − | − | N/A | N/A | N/A |
| P.433 | + | + | − | − | − | N/A | N/A | N/A |
| P.454 | + ^NC^ | − | − | + | + | N/A | N/A | N/A |
| P.465 | + | + | − | + | − | N/A | N/A | N/A |
| P.531 | +^W^ | − | − | − | − | N/A | N/A | N/A |
| P.662 | + | − | − | − | − | N/A | N/A | N/A |
| P.691 | + | − | − | − | + | N/A | N/A | N/A |
| P.747 | − | − | − | − | − | N/A | N/A | N/A |
| P.864 | + | − | − | − | − | N/A | N/A | N/A |

1: Results obtained by conventional PCR using outer primers F3 and B3.

2: Results obtained by LAMP reactions using F3, B3, FI, BIP, loopF and loopR primers.

3: Endophytic fungus isolated from *Buxus* ‘Green Gem’ foliage collected in 2013 from the Arnold Arboretum, Jamaica Plains, MA.

W indicates weak amplification.

NC indicates non-consistent amplifications.

N/A indicates information not available.

**Supplementary Table S2. List of LAMP primers evaluated in this study**

| **Primer sets/**  **approach** | **Primer^1^** | **Sequence (5’→3’) ^2^** | **Tm^3^** | **GC%^3^** |
| --- | --- | --- | --- | --- |
| **Unique regions** |  |  |  |  |
| **P.1** | F3 | TACACTAAGCGGCAGACT | 60.0 | 50.0 |
|  | B3 | AGTGCCTATTGAAGTGCTATG | 60.2 | 42.9 |
|  | FIP | CCAGGCCGGAATCATATCCCCCTTC  TAACCGCGAATCC |  |  |
|  | BIP | AATGCCGATCCTGAGATGCGCTCAA  GAGTAATCTCGCCATT |  |  |
|  | loopF | CTGCCCGTACCAATAACAGTA | 61.9 | 47.6 |
|  | loopR | ACGATGTCAGAAATAGCCTTCT | 61.6 | 40.9 |
|  |  |  |  |  |
| **P.12** | F3 | ACATCTAACCTAACAACAGAGC | 60.1 | 40.9 |
|  | B3 | GGAACATAATCGATGAGGAGAT | 59.5 | 40.9 |
|  | FIP | CCGATATATGCGGCGGTCCGAGCCG  ACAAGTATGGTG |  |  |
|  | BIP | ACAATTCATTTTTCGCCCTCGGGGGT  AGAATTGAATGATTCGC |  |  |
|  | loopF | ACCAGAATAATTGCCGCCTAA | 62.2 | 42.9 |
|  | loopR | CAAATATGGGACAACAATCGGT | 61.3 | 40.9 |
|  |  |  |  |  |
| **P.17** | F3 | ATGGTTGTGCTCCGTATG | 59.5 | 50.0 |
|  | B3 | AGCTTCGTCTCGATGACTA | 59.9 | 47.4 |
|  | FIP | ACAGGGAGCACAGTTTGACAATCTTC  ACATTGGCGCAATC |  |  |
|  | BIP | GTGGGTCATCTGTCGACTGTGCAACG  ACTATCCACAGGC |  |  |
|  | loopF | CGTCTAATCCGACCTGTCATT | 61.9 | 47.6 |
|  | loopR | GATCACCGACCATCCTGAC | 62.0 | 57.9 |
|  |  |  |  |  |
| **P.44A** | F3 | CAATGTCCCTCTGGATTGAG | 60.0 | 50.0 |
|  | B3 | CAACACGACAAGACCTCG | 60.4 | 55.6 |
|  | FIP | GCCAGACAGCGATGTGACATATGTCC  TGATCTTCAAGCATC |  |  |
|  | BIP | CAACACTCGCTTGCACCATCCATTGGT  AGGTGATGGATGG |  |  |
|  | loopF | ACACCACTTGAATCGTTCCA | 61.9 | 45 |
|  | loopR | CGAATTTCCCATGCTTGAGG | 61.6 | 50 |
|  |  |  |  |  |
| **P.44B** | F3 | GCTCAAGGCGTTCTTACTTA | 60.0 | 45.0 |
|  | B3 | TGCTGGATTATTCAGTGTCTC | 59.8 | 42.9 |
|  | FIP | AGGTAAGACAAGATTCCGCAGCCAAC  TCAAGGCACTCAGG |  |  |
|  | BIP | CCTCACTATCCACACCAGTGCGGTTGA  ATTTGCAGGAAGTG |  |  |
|  | loopF | AAGTTGCTCCTAGTTACTCGC | 62.1 | 47.6 |
|  | loopR | ACAGAAGGCTCCCTTTGAAG | 62.0 | 50.0 |
| **SNPs** |  |  |  |  |
| **P.1** | F3 | CAGGAGCCAGCAATTGAA | 60.0 | 50.0 |
|  | B3 | AACCGAGCATCGATTTCAA | 59.8 | 42.1 |
|  | FIP | ACGAAAGGCGAATGGGACAAAAGTATGAAGATGATGCTCCTG |  |  |
|  | BIP | GTTGTCGTGATCGATCGGAACTTTCCAAGCTGAATCTTACGG |  |  |
|  | loopF | ATCCCTGGGCAGTCTCAT | 62.2 | 55.6 |
|  | loopR | AGAACCACACTCGAACGC | 62.4 | 55.6 |

**Supplementary Table S1.** Continued

| **Primer sets/**  **approach** | **Primer^1^** | **Sequence (5’→3’) ^2^** | **Tm^3^** | **GC%^3^** |
| --- | --- | --- | --- | --- |
| **SNPs** |  |  |  |  |
| **P. 20** | F3 | CCATCGACTTCGACTTCG | 60.0 | 55.6 |
|  | B3 | GTGACACCAGCTGTGATC | 60.1 | 55.6 |
|  | FIP | ACAGCTTCGGTGAGTTCTGCATTATTGCCAGGAACGGAC |  |  |
|  | BIP | CTATTCAGCTCCTCACGCTTCCGGTCCAATGCTGAGGAAC |  |  |
|  | loopF | GCTGTGATTCCAACTCCGA | 62.1 | 52.6 |
|  | loopR | TGGCTGGCATCAATGTCC | 62.5 | 55.6 |
|  |  |  |  |  |
| **P.25-1** | F3 | CTTAAGTTCCTTGTCCCTCG | 60.0 | 50.0 |
|  | B3 | CGGTATTATCGGCATCATGT | 60.0 | 45.0 |
|  | FIP | GGACCATCGCTCGCAGTAGCTGACCAACGACCTTGTC |  |  |
|  | BIP | GGAACCAAGCCATCCGACATATAGACAGCTACAACGAGATTG |  |  |
|  | loopF | TTGATGATGTGGCTCTACGAG | 61.9 | 47.6 |
|  | loopR | CACGTCCCAGGATCTTGG | 61.9 | 61.1 |
| **Bioinformatics** |  |  |  |  |
| **P.119** | F3 | CATACCAAGTGCTCCAGC | 60.2 | 55.6 |
|  | B3 | CGTTGCAGAGTGAAATTTGG | 60.3 | 45.0 |
|  | FIP | GCCACCCCGTTCCAATAGAGGTTGCGAGCACTAATAACATAC |  |  |
|  | BIP | CTGATACGCAGTACTCCGTACAGTGCACTTGCATTAAACGAA |  |  |
|  | loopF | GCACGAGACTTTTGTGTTGG | 62.3 | 50.0 |
|  | loopR | TCCTGTGTTTCGGATGTTGT | 61.9 | 45.0 |
|  |  |  |  |  |
| **P.152** | F3 | AGCGGTGTATCGACTTATTTC | 60.2 | 42.9 |
|  | B3 | CGTACTAACCAACGACTCTC | 59.8 | 50.0 |
|  | FIP | CCATGCCGTCTCGTCTGTCCGTATTAGCATTGCGACCT |  |  |
|  | BIP | TTACGGCGCATGATCTCGGAAGAAGAGCAGCAGCATG |  |  |
|  | loopF | CCACGTTGGGGAAGAAGAA | 62.0 | 52.6 |
|  | loopR | TTTGCATCTCGTAGCCACT | 61.4 | 47.4 |
|  |  |  |  |  |
| **P.267** | F3 | TCGATGTGCTCTTCAACC | 59.4 | 50.0 |
|  | B3 | GTAGCGAGAGCGATGATG | 59.8 | 55.6 |
|  | FIP | CGGAGCAAAAGGGGCAGTGCTTCGTCAACATGGAGAG |  |  |
|  | BIP | CCGACGAGCCCAAGAGCTAAAGAGGTTCGTGATGTGC |  |  |
|  | loopF | AACTGGAAGTACAGCAGGC | 62.1 | 52.6 |
|  | loopR | CTTCTACTACGCCCTGCC | 61.6 | 61.1 |
|  |  |  |  |  |
| **P.267_13** | F3 | GACAAATGTTTCACCAGGATTC | 60.1 | 40.9 |
|  | B3 | TTACAAGTCAGGCGTTCG | 59.7 | 50.0 |
|  | FIP | TTTGTGCCTCTCGCTCGGTTAATGAGAGAGCCCTGACA |  |  |
|  | BIP | TTGTTCATTGTGCAGGTGGTCTAGAGAAGGCCAAGTAGCA |  |  |
|  | loopF | CTCGAGTCTCAATGCAAATCAC | 61.9 | 45.5 |
|  | loopR | CAGCAGCCTTCGACTTGA | 62.1 | 55.6 |
|  |  |  |  |  |

**Supplementary Table S1.** Continued

| **Primer sets/**  **approach** | **Primer^1^** | **Sequence (5’→3’) ^2^** | **Tm^3^** | **GC%^3^** |
| --- | --- | --- | --- | --- |
| **Bioinformatics** |  |  |  |  |
| **P.269** | F3 | GAGACAACCCTGTTCAACTT | 60.0 | 45.0 |
|  | B3 | GCTAATTGCAATGGCGAAG | 60.1 | 47.4 |
|  | FIP | TCTGGCACCTGCTTAACTTGGACGAGTTAACCTGAGTGGA |  |  |
|  | BIP | AGATGTGGCACAAGCGAGTCCGCATTGGAACCTCTAC |  |  |
|  | loopF | CTTTGGCAGCTTGGCAAC | 62.4 | 55.6 |
|  | loopR | AATAACCACTGCGATATCCGT | 61.7 | 42.9 |
|  |  |  |  |  |
| **P.304** | F3 | GCAGCCTTTCTTAATACGAGA | 60.2 | 42.9 |
|  | B3 | CGTGATTGCGAAGATGATTG | 59.9 | 45.0 |
|  | FIP | TTCCAGTTCGAGGGTCCCACCATCAGCTGTCTCAACG |  |  |
|  | BIP | CCAGCTTAGTCATCGCATCACAAACAGGAAACAATGGGTCC |  |  |
|  | loopF | CCAGCTAGTTGTAACAGGCA | 62.1 | 50.0 |
|  | loopR | CGGGACTCCAATATCACAACT | 61.9 | 47.6 |
|  |  |  |  |  |
| **P.329** | F3 | ATGCGGCCAATGTAGATG | 59.9 | 50.0 |
|  | B3 | TGAGGAGAAGGTTGAGGAG | 60.1 | 52.6 |
|  | FIP | CCAGGACGTTGGCAAGATCCAGACTCTTCGTCAGAGGC |  |  |
|  | BIP | GTCATGTCCTCGTCGCCAGCTGAGGATGAGGAGGAGG |  |  |
|  | loopF | CAAGGTGTCCAAGGAGATCAA | 62.0 | 47.6 |
|  | loopR | GTCGAGCTCCGTCACAAG | 62.5 | 61.1 |
|  |  |  |  |  |
| **P.344** | F3 | CGAGACCATGTTTGACGAA | 60.2 | 47.4 |
|  | B3 | GCCACTTTACAAGCAGTTTAG | 59.7 | 42.9 |
|  | FIP | CTGCGTCTCACTAGCAACGAGAATATTTGCCAGGCTCGG |  |  |
|  | BIP | AACTCCACTAACAGCACTCGTGCGGCGACATTGCTACTATT |  |  |
|  | loopF | TTGGCCTTCGTCTGAACC | 62.0 | 55.6 |
|  | loopR | CCCTGGGGTTGAAGTTTCA | 61.9 | 52.6 |
|  |  |  |  |  |
| **P.379** | F3 | ACCTCGGTAATAGCCTCC | 59.9 | 55.6 |
|  | B3 | TCCCTTAACACCGAAGTCT | 60.0 | 47.4 |
|  | FIP | TGAGCAGTGCAACACCAGGGAATGCACCCGTTCAGAA |  |  |
|  | BIP | GCTGCGCTCGAGGAAATTGCACGTCTGCAAGAGACAG |  |  |
|  | loopF | TAGCGGCGCAAAGCATTA | 62.6 | 50.0 |
|  | loopR | ACAGATCAGCAAGGGATTCC | 61.9 | 50.0 |
|  |  |  |  |  |
| **P.381** | F3 | GAGCGTTGGAAAAGGGAT | 59.7 | 50.0 |
|  | B3 | AGACCAGAAGATCAAGCAATAG | 59.9 | 40.9 |
|  | FIP | ACTGTCGCACAGACAAACACACAGCCAATCGGGTTGTAG |  |  |
|  | BIP | TGATCCACCTTCTTGTCGCTGCCTGGGACAAATGTGTGT |  |  |
|  | loopF | AAAGCGAAGCATTCAGGC | 61.1 | 50.0 |
|  | loopR | ACACCGATAATACTGTCGCAA | 61.9 | 42.9 |
|  |  |  |  |  |

**Supplementary Table S1.** Continued

| **Primer sets/**  **approach** | **Primer^1^** | **Sequence (5’→3’) ^2^** | **Tm^3^** | **GC%^3^** |
| --- | --- | --- | --- | --- |
| **Bioinformatics** |  |  |  |  |
| **P.393** | F3 | AATCTACCAGCCGGATCA | 59.8 | 50.0 |
|  | B3 | TTGTGATCTCGTGTTGGAAG | 60.2 | 45.0 |
|  | FIP | GCTCGCAGGGCTTCTCAATGTCGAGTTCGAGTTCCT |  |  |
|  | BIP | CCAACAACAGCGTGTCGCTGACTTACACAGAGGCGTA |  |  |
|  | loopF | GTCACGGTGTGGTTCTGG | 62.7 | 61.1 |
|  | loopR | GGCCATGCAGGTAATGGT | 62.2 | 55.6 |
|  |  |  |  |  |
| **P.402** | F3 | TACCTGTCGTCCACGAAC | 61.1 | 55.6 |
|  | B3 | ATGGTCTGGATATCAATGCG | 59.9 | 45.0 |
|  | FIP | TCACGCAGCGCGGTATTCGCTGTCATCCGCAGTAAG |  |  |
|  | BIP | CTACTGGTCGCACGAGGTGCCTCAATGACATCACGCT |  |  |
|  | loopF | ACAATACCTAGTGCGCTCAG | 61.9 | 50.0 |
|  | loopR | GATGAGCGAGGTCATGGAG | 62.0 | 57.9 |
|  |  |  |  |  |
| **P.420** | F3 | CCTCCTCTTCGTTGTTGG | 59.7 | 55.6 |
|  | B3 | CCAAGAACGTGTCCTGAC | 60.0 | 55.6 |
|  | FIP | GCCGACATTCCTTCGCAGATCCTGAAACCTTGTTGTCAG |  |  |
|  | BIP | GGACTCGGTTTCATGCCTGGACAAGAAGAAGAAGAACGCA |  |  |
|  | loopF | TTGCTGGTCCCCGAATTG | 62.4 | 55.6 |
|  | loopR | GGAGGGGCATCCTTGTTT | 61.8 | 55.6 |
|  |  |  |  |  |
| **P.433** | F3 | TTGCCTGTGATCTGATTAGAG | 59.5 | 42.9 |
|  | B3 | TAGTGCACGATGCTGATTC | 60.0 | 47.4 |
|  | FIP | CTTGAACGACCACCTCACCAAACATTGTCTTCTGGCTTCG |  |  |
|  | BIP | ACGATGGCTTCGTGCCTTAGTCCGTTTGAGAAGGAGGT |  |  |
|  | loopF | GGCTCTCCGATATCTCAAGAAG | 62.1 | 50.0 |
|  | loopR | TTCCAATCAGGAGGTTCTTCTC | 61.8 | 45.5 |
|  |  |  |  |  |
| **P.454** | F3 | TAATGACCACAACCACAGC | 60.1 | 47.4 |
|  | B3 | CACAGCGATTGATGACGA | 59.9 | 50.0 |
|  | FIP | GCACGCACATCATGAGCTGTCAGAGACAGCTCCTTGG |  |  |
|  | BIP | GTAATGGATTGCCCATGCTGCGGATGGAATGCACGTCATA |  |  |
|  | loopF | CGGCTCAGTCCAATGGTATT | 62.2 | 50 |
|  | loopR | GCCATCACAAGATGAAGGC | 61.3 | 52.6 |
|  |  |  |  |  |
| **P.465** | F3 | TGTGCCGATAGGTTATTGC | 60.0 | 47.4 |
|  | B3 | ATCGGACATCGAGGAGAG | 60.0 | 55.6 |
|  | FIP | GAGAGGATAGAGGAGCTGGAGGTGAGCTTGCTGACATCATC |  |  |
|  | BIP | CTTGTCTCGCCGTAGCCTGAGGAGCAATACCAGAACCT |  |  |
|  | loopF | ATGAGGAACTCGAGAACGC | 61.9 | 52.6 |
|  | loopR | CGCTCAGTGTCGCCTTAA | 62.1 | 55.6 |
|  |  |  |  |  |

**Supplementary Table S1.** Continued

| **Primer sets/**  **approach** | **Primer^1^** | **Sequence (5’→3’) ^2^** | **Tm^3^** | **GC%^3^** |
| --- | --- | --- | --- | --- |
| **Bioinformatics** |  |  |  |  |
| **P.531** | F3 | CAGCTATGGAACGCCATAA | 59.7 | 47.4 |
|  | B3 | ACAACTCGGATAGCGGTA | 59.8 | 50.0 |
|  | FIP | CGCAGCTTTGCCCACTTTGGACTACCTCAAGGTTCGC |  |  |
|  | BIP | GCAGCTGGAGAAGTCGAGTATGGCAGCTTCCGTTAGTATTGT |  |  |
|  | loopF | AATTCGTCCTGGCTGCTC | 62.2 | 55.6 |
|  | loopR | GGAGAAACAAGCTACATGCG | 61.6 | 50.0 |
|  |  |  |  |  |
| **P.662** | F3 | ATCTCATGCAAGTGTCGG | 59.5 | 50.0 |
|  | B3 | GCGCCAATGGTGTTAATTATG | 60.6 | 42.9 |
|  | FIP | ATCGCGTGGTGACTTGGCACCAACATCATCACGAGC |  |  |
|  | BIP | CATCATCTCTGGATCCAGCCAATGAGCACCACTTCGATAAC |  |  |
|  | loopF | CAGGGCTCAGACTGTGTG | 62.1 | 61.1 |
|  | loopR | CCTCCTCGTCAGCATCTTC | 62.0 | 57.9 |
|  |  |  |  |  |
| **P.691** | F3 | CGGGAGTCTTAGACTAAGAGAT | 60.3 | 45.5 |
|  | B3 | TTAAGCATGCACTGGTCG | 60.1 | 50.0 |
|  | FIP | GCTTCCCGCTTCTGCTACACAGCTCTATCCGATCCCA |  |  |
|  | BIP | CACCAAACACTCCCACCCTCGGCTCCGTAATTGCATATAGT |  |  |
|  | loopF | TGTCATGTCGCAGATAGTCG | 62.0 | 50.0 |
|  | loopR | TCAGAATAGCCAAGGCGATC | 62.0 | 50.0 |
|  |  |  |  |  |
| **P.747** | F3 | TCTCCTGTTATCCTCACAACTA | 60.0 | 40.9 |
|  | B3 | GCGTGAATGCTGTGGTAT | 60.2 | 50.0 |
|  | FIP | CGTTCCACGAGTCGATGAGGGGCAAGGCTAACAAGGAA |  |  |
|  | BIP | CGCCTTGGCCGAGTACAACGAATCGTCAGTGAGATCG |  |  |
|  | loopF | CACACCTCCTCATGGCTG | 62.2 | 61.1 |
|  | loopR | GACACCAGCCTTCAGTCTC | 62.1 | 57.9 |
|  |  |  |  |  |
| **P.864** | F3 | TCGGTGTTGACGAGGTAT | 60.0 | 50.0 |
|  | B3 | CGAGATAGCCAAGCGAATT | 60.1 | 47.4 |
|  | FIP | TCGCCGTCTGCCATGATATTCACACCAGGCGTAGTAGTT |  |  |
|  | BIP | TGCCCTGGCTCCTGTACTGGCTTCATCGTCATACCAA |  |  |
|  | loopF | AAGGCATTATCACAGTCTCAGG | 62.2 | 45.5 |
|  | loopR | GACGGTGATTCCACAATGTTG | 62.1 | 47.6 |

^1^: F3: Forward outer; B3: reverse outer; FIP and BIP: inner LAMP primers; loopF and loopR: forward and reverse loop primers.
